# Genomic Heritability and Genome-Wide Association Studies of Plasma Metabolites in Crossbred Beef Cattle

**DOI:** 10.3389/fgene.2020.538600

**Published:** 2020-09-24

**Authors:** Jiyuan Li, Everestus C. Akanno, Tiago S. Valente, Mohammed Abo-Ismail, Brian K. Karisa, Zhiquan Wang, Graham S. Plastow

**Affiliations:** ^1^Livestock Gentec, Department of Agriculture, Food and Nutritional Science, Faculty of Agricultural, Life and Environmental Sciences, University of Alberta, Edmonton, AB, Canada; ^2^Department of Animal Science, Ethology and Animal Ecology Research Group, São Paulo State University, Jaboticabal, Brazil; ^3^Department of Animal Science, College of Agriculture, Food and Environmental Sciences, California Polytechnic State University, San Luis Obispo, CA, United States; ^4^Ministry of Agriculture and Forestry, Edmonton, AB, Canada

**Keywords:** candidate genes, crossbred beef cattle, functional enrichment analyses, metabolomics, single-step GBLUP

## Abstract

Metabolites, substrates or products of metabolic processes, are involved in many biological functions, such as energy metabolism, signaling, stimulatory and inhibitory effects on enzymes and immunological defense. Metabolomic phenotypes are influenced by combination of genetic and environmental effects allowing for metabolome-genome-wide association studies (mGWAS) as a powerful tool to investigate the relationship between these phenotypes and genetic variants. The objectives of this study were to estimate genomic heritability and perform mGWAS and *in silico* functional enrichment analyses for a set of plasma metabolites in Canadian crossbred beef cattle. Thirty-three plasma metabolites and 45,266 single nucleotide polymorphisms (SNPs) were available for 475 animals. Genomic heritability for all metabolites was estimated using genomic best linear unbiased prediction (GBLUP) including genomic breed composition as covariates in the model. A single-step GBLUP implemented in BLUPF90 programs was used to determine SNP *P* values and the proportion of genetic variance explained by SNP windows containing 10 consecutive SNPs. The top 10 SNP windows that explained the largest genetic variation for each metabolite were identified and mapped to detect corresponding candidate genes. Functional enrichment analyses were performed on metabolites and their candidate genes using the Ingenuity Pathway Analysis software. Eleven metabolites showed low to moderate heritability that ranged from 0.09 ± 0.15 to 0.36 ± 0.15, while heritability estimates for 22 metabolites were zero or negligible. This result indicates that while variations in 11 metabolites were due to genetic variants, the majority are largely influenced by environment. Three significant SNP associations were detected for betaine (rs109862186), L-alanine (rs81117935), and L-lactic acid (rs42009425) based on Bonferroni correction for multiple testing (family wise error rate <0.05). The SNP rs81117935 was found to be located within the *Catenin Alpha 2* gene (*CTNNA2*) showing a possible association with the regulation of L-alanine concentration. Other candidate genes were identified based on additive genetic variance explained by SNP windows of 10 consecutive SNPs. The observed heritability estimates and the candidate genes and networks identified in this study will serve as baseline information for research into the utilization of plasma metabolites for genetic improvement of crossbred beef cattle.

## Introduction

The metabolic phenotype (or “metabotype”) is a characteristic metabolite profile that depends on the interactions between genetic and environmental effects. Commonly, the metabolic phenotype of an individual is measured from easily accessible biofluids such as urine or blood (Nicholson and Lindon, 2008). Additionally, blood metabolites have been shown to be powerful tools for the indication of the nutritional and health status of humans and animals. For example, in humans, several blood metabolites have been identified as biomarkers for diseases (López-López et al., 2018). In livestock species, associations between metabolites and economically important traits such as feed efficiency (Karisa et al., 2014), growth performance (Widmann et al., 2013), and animal health (Montgomery et al., 2009) have been reported.

Metabolome-genome-wide association study (mGWAS) is a powerful tool for identifying genetic variants underlying metabolic phenotypes and provides new opportunities to decipher the genetic basis of metabolic phenotypes. Importantly, mGWAS detect genetic variants that are functionally associated with metabolic phenotype variation. For example, previous studies have reported that single nucleotide polymorphisms (SNPs) in the *glutamine synthase 2* gene (*GLS2*) were associated with glutamine in human serum, which provides a potential biological association, as the enzyme GLS2 catalyzes the hydrolysis of glutamine (Suhre et al., 2011; Kettunen et al., 2012). Furthermore, genome-wide hits with unknown gene function offer an opportunity to infer novel biological mechanisms of the SNP-metabolite association. For instance, Suhre et al. (2011) experimentally studied the association of the SNP rs7094971 inside the *solute carrier family 16*, *member 9* gene (*SLC16A9*) with carnitine and validated that the hitherto uncharacterized protein was indeed a carnitine transporter in *Xenopus* oocytes. Additionally, as metabolites lie between genomic and external phenotypes, they could explain the variation of external phenotypes by revealing biological mechanisms underlying the associations between them. Recent application of GWAS have successfully uncovered genetic variants that contribute to variation in both the external phenotype (e.g., type 2 diabetes) and the metabolic phenotype (e.g., fasting glucose levels) (Stranger et al., 2011).

Due to the rapidly growing number of candidate biomarkers and the increasing role of metabolites in genetic studies, the knowledge of the genetic basis of metabolites is therefore a prerequisite to evaluate new biomarkers and dissect the genetic architecture of metabolites. Until now, however, knowledge regarding the genetic level of metabolites in beef cattle has been limited. Thus, the objectives of this study were to estimate genomic heritability of 33 plasma metabolites in crossbred beef cattle, to identify genetic variants, genomic regions and candidate genes associated with metabolite variation, and to understand the biological functions and gene networks linked to these associations.

## Materials and Methods

### Animal, Blood Samples and Nuclear Magnetic Resonance (NMR) Spectroscopy

All management and procedures involving live animals, where applicable, conformed to the guidelines outlined by the Canadian Council on Animal Care (1993); otherwise, existing data sets from the various Canadian research herds were used.

The dataset used in this study was obtained from the Phenomic Gap Project (McKeown et al., 2013). This project started in 2008 aiming to generate feed efficiency, carcass and meat quality phenotypes as well as genomic information for Canadian crossbred beef animals as previously described by Akanno et al. (2014). A total of 475 Canadian multibreed composite and crossbred beef cattle was used in this study. The animals comprised of bulls, slaughter steers, slaughter heifers and replacement heifers submitted to a feedlot feeding test from 2009 to 2012 and the test groups were labeled as contemporary groups. The population structure consisted of Beefbooster composite breed (*n* = 224) which is predominantly Charolais-based with infusion of Holstein, Maine Anjou, and Chianina^1^, Hereford-Angus (*n* = 181) crosses, Charolais (*n* = 68), and Angus (*n* = 2).

Blood samples were collected in EDTA tubes from each animal by jugular venipuncture on the first day of the feedlot feeding test and immediately frozen at −80°C which is considered appropriate for storage. Our assumption is that all samples were affected equally by the freezing process if at all. Therefore, although the metabolite profiles may not be the same as those obtained from fresh samples, the freezing process should not be a source of variation for this study since all samples were frozen the same way according to best practice. Frozen blood samples were sent to the Metabolomics Innovation Center at University of Alberta, AB, Canada in 2014 for analysis. The variation in time of sample collection is expected to be captured under the “contemporary group” variable applied in subsequent statistical analysis. Each frozen sample was thawed at room temperature then centrifuged at 10,000 rpm for 10 min to separate the plasma then filtered through 3 kDa molecular weight cut-off filters (Merck Millipore Ltd., Darmstadt, Germany) to remove macromolecules, including lipids and proteins. As the filter tube manufacturer treats the filter membranes with glycerol as a preservative, filters were washed and centrifuged five times before use. Samples made up of less than 570 μl after filtration were diluted with HPLC water to ensure adequate volume for NMR acquisition. A total of 5 mm NMR tube (New Era Enterprises Inc., Vineland, NJ, United States) contained a total of 700 μl of total volume of 570 μl filtered serum, 60 μl DSS and 70 μl D2O. This mixture was vortexed and centrifuged shortly before it was transferred to an NMR tube for data acquisition. All metabolite concentrations obtained were adjusted by appropriate factors to account for the above dilutions, and represent the contents of the filtered samples, not the contents of the NMR tube.

Spectra were acquired on a 500MHz VNMRS spectrometer equipped with a 5mm cold probe (Agilent Technologies, Santa Clara, CA, United States). The pulse sequence used was a 1D-noesy with a 990 ms presaturation on water and a 4 s acquisition period. Spectra were collected with 256 transients and four steady-state scans at 298K.

Spectra were zero filled to 64k points and Fourier transformed. Spectral phasing was performed on the spectra along with baseline correction. In total, 33 metabolites were identified and quantified with a targeted profiling approach using the Profiler and Library Manager modules in the same software which contains a total of 304 metabolites. Each spectrum was peer reviewed by a separate analyst and a final review pass was done on all of the spectra before exporting concentration results. Concentration measurements were adjusted to report metabolite concentrations after the filtration of the samples.

### Genotyping, Quality Control and Prediction of Genomic Breed Composition

Animals were genotyped using Illumina BovineSNP50 v2 BeadChip (Illumina Inc., San Diego, CA, United States) at Delta Genomics, Edmonton, AB, Canada. The genotypes were coded as 0, 1, and 2 and quality control was performed using the Synbreed package (Wimmer et al., 2012) in R statistical software. All markers on sex chromosomes and autosomal markers with minor allele frequency <1%, call rate <90%, and severe departure from Hardy-Weinberg equilibrium (*P* < 10^–5^) were removed. Missing genotypes were imputed using Synbreed package. After quality control, 45,266 SNPs on 29 bovine autosomes for 475 individuals remained and were used for this study.

**FIGURE 1:**
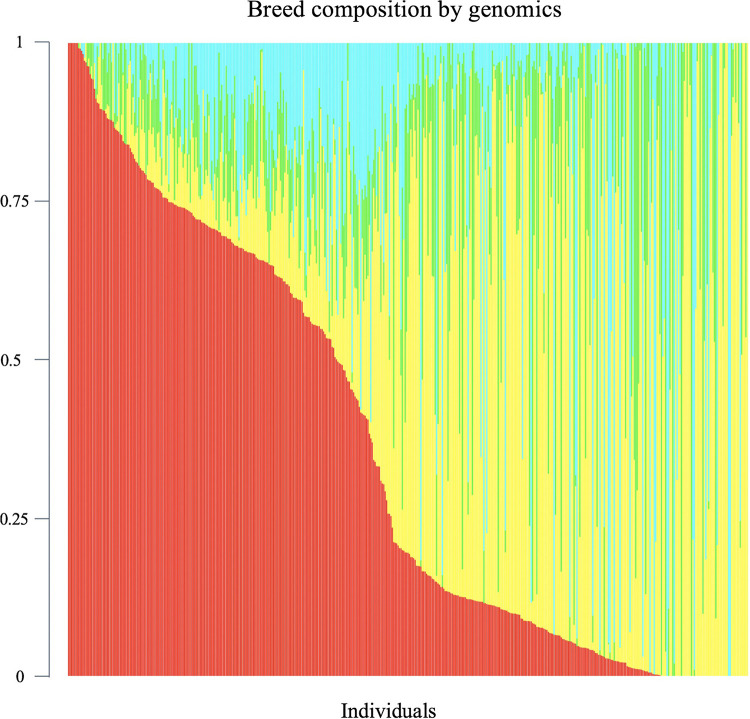
Distribution of predicted genomic breed composition of crossbred beef cattle population (*n* = 475). Beefbooster is red, Angus is yellow, Hereford is green, Charolais is blue.

Genomic breed composition was predicted for all individuals using ADMIXTURE software (Alexander et al., 2009). To predict breed composition for each animal, a 10-fold cross-validation procedure was performed to find the best possible number of ancestors or breeds (*K* value). The value of *K* = 4 was chosen because it had the smallest cross-validation error and yielded the most accurate breed composition prediction based on prior knowledge. The four postulated ancestral breeds were Hereford, Angus, Charolais and Beefbooster TX line. The distribution of predicted genomic breed composition is shown in Figure 1. Estimates of genomic breed composition were fitted as covariates in the various statistical models to correct for population stratification and breed effects.

### Phenotypic Quality Control

Phenotypic records included 33 plasma metabolite concentrations quantified from blood samples of 475 animals. A linear regression model implemented in R statistical software was used to assess the significance of all systematic effects associated with variation in plasma metabolites. Fixed factors found to be significant (*P* <0.05) included contemporary groups (herd and birth year), animal type (bulls, slaughter steers, slaughter heifers, and replacement heifers) and genomic breed composition. These factors were subsequently included in the mixed model used for estimating heritability and GWAS. Contemporary group and animal type were fitted in the model as fixed class effect whereas breed fractions were fitted as fixed covariates. Residual values of the linear regression model were checked and those residuals with more or less than 3 standard deviations from the mean of residuals were considered as outliers and the associated records were excluded. The distribution of residuals after excluding outliers was close to a normal distribution (i.e., a bell shape without a big tail). The summary statistics of all metabolites after phenotypic quality control are given in Table 1. In general, the concentration of plasma metabolites ranged from 20.72 μM (L-methionine) to 5,024.04 μM (L-lactic acid), on average.

**TABLE 1:** Descriptive statistics for 33 plasma metabolites: number of animals per metabolite (*n*), mean, standard deviation (SD), coefficient of variation (CV), minimum (Min.) and maximum (Max.).

Trait	*n*	Mean	SD	CV	Min.	Max.
1-Methylhistidine	435	56.26	22.71	0.40	15.34	136.31
2-Hydroxybutyrate	460	41.23	17.02	0.41	12.26	94.48
Acetic acid	462	264.60	256.05	0.97	33.40	2,056.21
Betaine	448	111.67	52.97	0.47	29.62	298.33
Creatine	451	127.59	44.39	0.35	41.98	262.67
Citric acid	448	120.27	65.38	0.54	15.61	338.45
Choline	456	346.37	173.98	0.50	61.35	960.08
Ethanol	404	61.38	84.91	1.38	13.53	560.94
D-Glucose	452	837.40	692.11	0.83	68.42	3,731.80
Glycine	451	378.65	162.32	0.43	90.38	896.70
Glycerol	452	511.10	354.71	0.69	15.68	1,532.64
Fumaric acid	300	23.85	8.48	0.36	10.75	66.11
Formic acid	454	30.34	28.25	0.93	9.46	370.87
L-Tyrosine	475	65.51	19.32	0.29	22.88	119.90
L-Phenylalanine	454	67.54	19.54	0.29	27.53	125.61
L-Alanine	446	390.34	148.99	0.38	104.46	852.47
L-Proline	465	129.58	41.02	0.32	42.09	257.82
L-Isoleucine	465	52.85	19.88	0.38	15.11	120.63
L-Histidine	450	76.09	28.57	0.38	23.35	150.45
Lysine	460	70.34	26.19	0.37	15.24	154.49
L-Lactic acid	450	5,024.04	2,790.01	0.56	885.17	15,976.05
Pyruvic acid	321	87.56	81.42	0.93	14.23	395.75
Succinic acid	448	58.47	34.46	0.59	14.86	280.58
3-Hydroxybutyric acid	457	86.65	41.66	0.48	18.29	272.70
Creatinine	451	132.14	57.85	0.44	30.77	308.61
L-Glutamine	441	58.97	23.00	0.39	14.35	119.97
L-Leucine	475	93.08	39.48	0.42	25.63	302.17
L-Methionine	193	20.72	4.49	0.22	12.08	33.77
3-Hydroxyisovaleric acid	155	32.38	13.02	0.40	11.70	79.06
L-Valine	454	147.16	49.58	0.34	49.88	313.97
Acetone	260	35.97	19.84	0.55	12.47	125.08
Methanol	447	135.47	76.28	0.56	31.35	383.19
Dimethyl sulfone	449	46.86	19.41	0.41	15.31	128.60

*Unit:μM.*

### Variance Components and Heritability Estimation

Variance components and heritability of 33 metabolites were estimated using a single-trait animal model and genomic relationship matrix. The genomic relationship matrix was constructed based on total filtered SNP markers (i.e., 45,266 SNPs) and using one of VanRaden’s formulations *ZZ*′/2∑*p*_*i*_(1−*p*_*i*_), where *Z* contains centered genotypes codes and *p*_*i*_ is the minor allele frequency for locus *i* (VanRaden, 2008). The following mixed effect model (1) implemented in ASReml version 4.1 (Gilmour et al., 2015) was applied:

(1) y=X⁢b+W⁢a+e

Where *y* is a vector of phenotypic observation; *X* is the design matrix that relates the fixed effects to the observation and *b* is a vector of fixed effects of contemporary groups, animal type and genomic breed composition. *W* is a design matrix relating observations to random animal genetic effects; *a* is a vector of random additive polygenic effects that is assumed to be normally distributed as: $a∼N\,\left(0,G\,\sigma_{a}^{2}\right)$, where*G* is genomic relationship matrix and $\sigma_{a}^{2}$ is the additive genetic variance, *e* is a vector of random residual effects that is assumed to be normally distributed as $e∼N\,\left(0,I\,\sigma_{e}^{2}\right)$, with *I* being an identity matrix and $\sigma_{e}^{2}$ is the residual error variance.

### Metabolome-Genome-Wide Association Study

The genomic heritability obtained from model (1) was used to screen all metabolites for metabolome genome wide association analyses. Metabolites with zero or near zero heritability were excluded from mGWAS. Here, the SNP *P* values for 11 metabolites with non-zero heritability were determined using a single-step genomic BLUP (ssGBLUP) approach as described by Aguilar et al. (2019) and followed by the estimation of the proportion of additive variance explained by 10 consecutive SNP windows using a Weighted ssGBLUP (WssGBLUP) approach (Wang et al., 2012). Both approaches were implemented in the BLUPF90 programs (Misztal et al., 2002). The mGWAS model used was similar to model (1) above except that *a* was assumed to follow $N\,\left(0,H\,\sigma_{a}^{2}\right)$, where *H* is the matrix that combines genomic and pedigree information (Aguilar et al., 2010). The inverse of *H* for mixed model equations is:

$$H^{-1}=A^{-1}+\left[\begin{array}{cc} 0 & 0\\ 0 & G^{-1}-A_{22}^{-1} \end{array}\right]$$

*A* is the pedigree-based numerator relationship matrix for all animals, *A*_22_ is the numerator relationship matrix for genotyped animals, and matrix *G* is the genomic relationship matrix, where *G* was weighted as described by Wang et al. (2012) for the WssGBLUP method.

A rejection threshold based on Bonferroni correction for multiple testing (0.05/45,266) was applied, which is equal to 5.96 in the −log10 scale. The quantile–quantile (Q–Q) plots of *P* values for each SNP were used to compare observed distributions of −log (*P* value) to the expected distribution under the null hypothesis for each metabolite. Manhattan plots of *P* values for each SNP were also used to illustrate significant associations at the level of each chromosome for the metabolites. All plots were completed using the R package qqman (Turner, 2014).

### Candidate Gene Identification

To identify a candidate gene, the surrounding region of each significant SNP was surveyed by expanding 100-kbp upstream and downstream, respectively. The 200-kbp region was defined based on the average linkage disequilibrium (*r*^2^) between pairs of syntenic SNPs within this distance which is known to be 0.20 in a related beef cattle population (Lu et al., 2012).

Further, additional candidate genes associated with the top 10 SNP windows that explained the largest proportion of genetic variance for each metabolite from the WssGBLUP approach were identified. Positional candidate genes within 200-kbp regions and those inside the top 10 SNP windows were mapped on *Bos taurus* genome view in Biomart available at the Ensembl database UMD 3.1 version (Zerbino et al., 2018). The functions of all identified genes were manually searched from the literature to see if they had a previously identified relationship with the associated metabolites under investigation.

### Analysis of Least Square Means for Significant SNP

The least square mean of SNPs significantly associated with metabolites were assessed based on model (2) and implemented in R where applicable, to see how different allele combinations for these SNPs resulted in observed differences in the metabolite concentration.

y=Xb+SNP+e                   (2)

Where *y*,*X*,*b*, and *e* are the same as in model (1) and (2); *SNP* is a vector of genotype class 0, 1 and 2 fitted as a classification factor.

### Functional Enrichment Analyses

The interpretation of mGWAS using metabolite concentrations as the target phenotype is a complicated task, because their concentrations are influenced indirectly by mRNA and protein expression as well as directly by several environmental effects. Pathway analysis using prior knowledge improves the interpretation of mGWAS data and provides insight from the genetics of biochemical conversions and biological functions. Functional analyses for the genes associated with each metabolite were performed using Ingenuity Pathway Analysis software^2^ (IPA). Several lists including metabolites (PubChem CID) and candidate genes (Bovine Entrez gene IDs) in Supplementary Table S1 were imported in IPA for biological function analysis and network construction. Biological functions were considered significantly enriched if the *P* value for the overlap comparison test between the input list and the knowledge base of IPA for a given biological function was less than 0.05. Identification of significant pathways in biological processes was described in detail by Calvano et al. (2005). The analysis was performed following IPA default setting and parameters were set to allow the network to show indirect relationships for the imported metabolite and gene lists. Indirect relationships assist in the identification of other metabolites/genes that were not among the ones in the input list but may be associated with them based on the IPA biological reference. In addition, the resulting gene networks are scored and then sorted based on the score not based on *P* value, as multiple testing for this sort of analysis is not feasible.

## Results

### Heritability Estimates

Eleven metabolites showed low to moderate heritability that ranged from 0.09 ± 0.15 (succinic acid) to 0.36 ± 0.15 (choline), while heritability estimates for 22 metabolites were zero or negligible. Table 2 shows the results of all metabolites with heritability.

**TABLE 2:** Estimates of additive variance ($\sigma_{a}^{2}$), residual variance ($\sigma_{e}^{2}$), heritability (*h*^2^) and their standard error (*SE*) for 11 plasma metabolites^*a*^.

Trait	$\sigma_{a}^{2}$	$\sigma_{e}^{2}$	*h*^2^	*SE*
Choline	6,598.90	11,545.80	0.36	0.15
Creatinine	1,051.67	1,947.73	0.35	0.17
Betaine	402.10	783.09	0.34	0.16
Pyruvic acid	1,027.32	2,007.84	0.34	0.24
L-Lactic acid	639,240	2,268,490	0.22	0.16
Citric acid	477.13	1,719.37	0.22	0.15
Creatine	160.55	843.99	0.16	0.15
D-Glucose	17,497.10	100,579.00	0.15	0.14
Acetone	29.39	185.01	0.14	0.21
L-Alanine	768.05	7,824.22	0.09	0.13
Succinic acid	78.47	838.28	0.09	0.15

*^*a*^Metabolites with zero or near zero heritability estimates were not listed.*

### SNP Association, Candidate Genes and Genetic Effects

**FIGURE 2:**
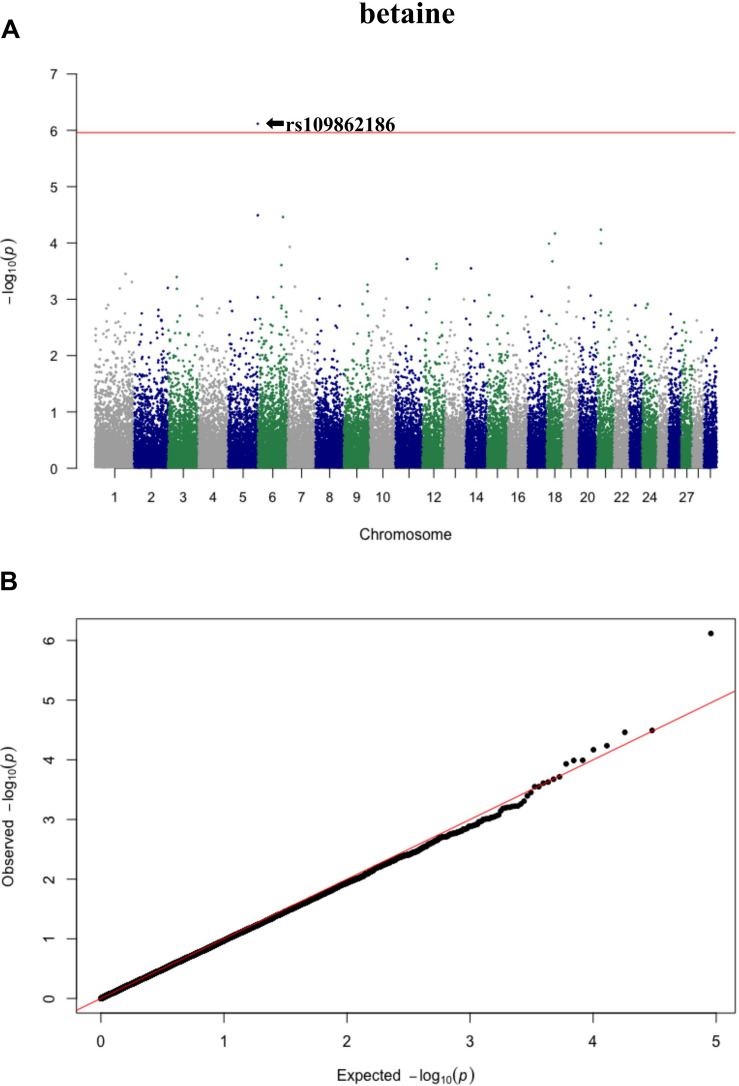
Manhattan plot **(A)** and QQ plot **(B)** for betaine, significant SNPs were determined by Bonferroni correction (red line).

**FIGURE 3:**
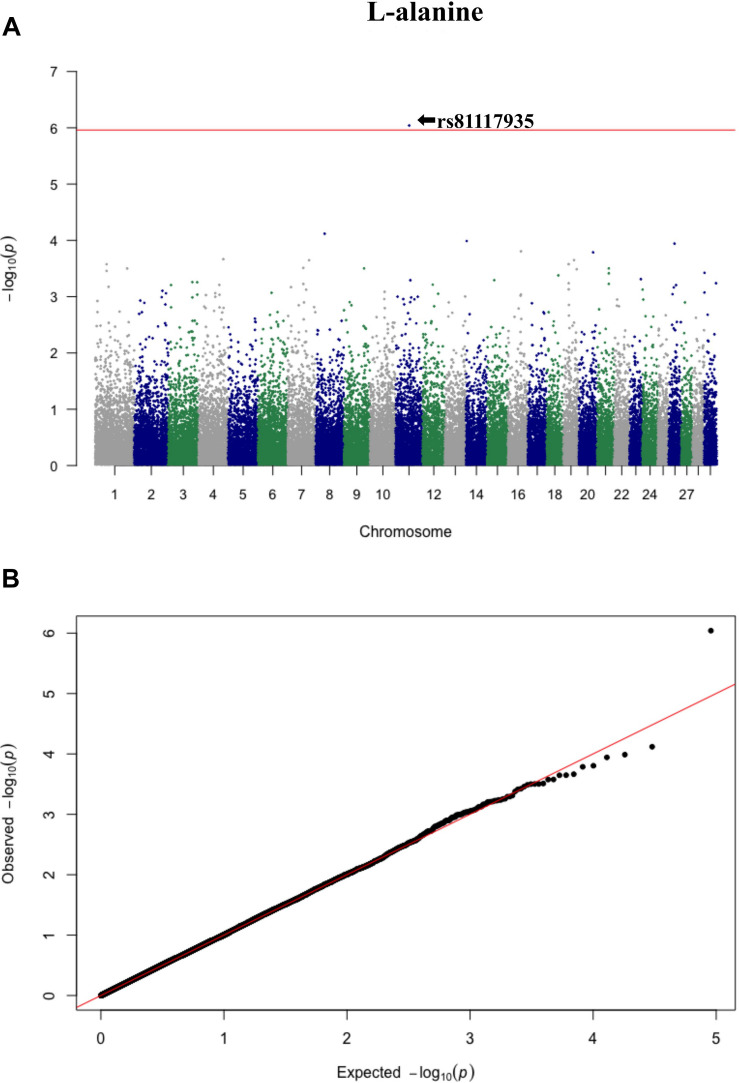
Manhattan plot **(A)** and QQ plot **(B)** for L-alanine, significant SNPs were determined by Bonferroni correction (red line).

**FIGURE 4:**
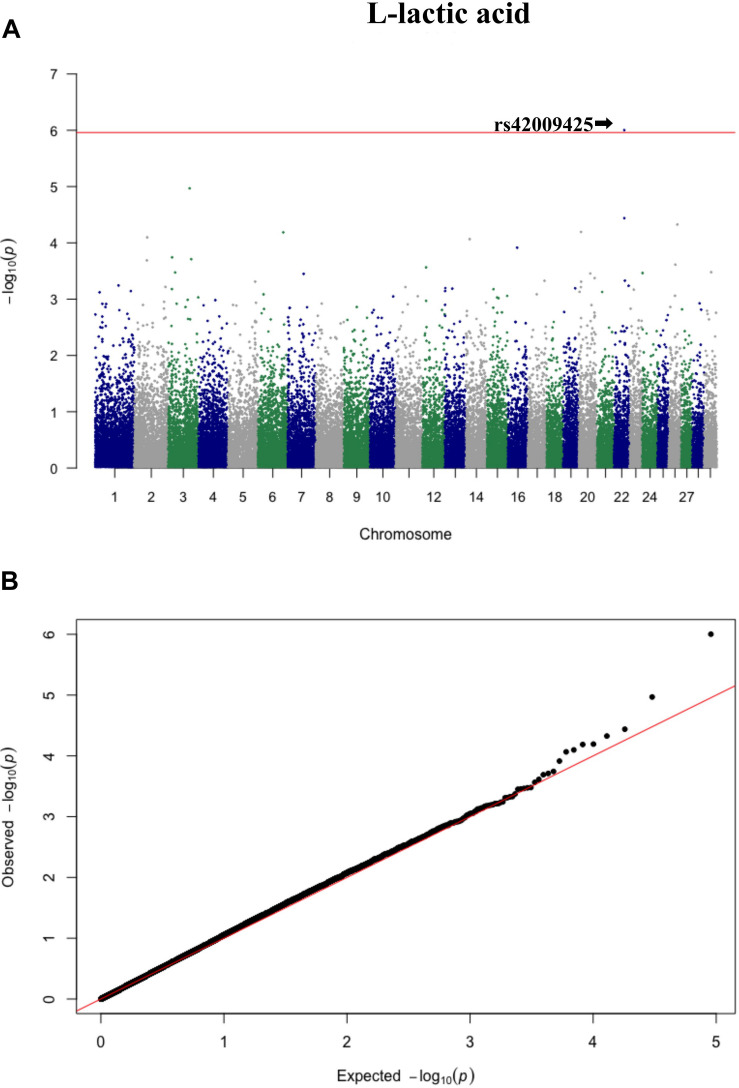
Manhattan plot **(A)** and QQ plot **(B)** for L-lactic acid, significant SNPs were determined by Bonferroni correction (red line).

Three significant SNP associations were detected for betaine (rs109862186), L-alanine (rs81117935), and L-lactic acid (rs42009425) based on Bonferroni correction for multiple testing (family wise error rate <0.05) (Table 3 and Figures 2–4). The SNPs were located on chromosome 5, 11, and 22, respectively. The SNP rs81117935 was found within the *catenin alpha 2* gene (*CTNNA2*), while the other two SNPs were not mapped to any known candidate gene (Table 4).

**TABLE 3:** SNPs significantly associated with metabolites: chromosome (Chr), position of SNP on chromosome (bp), minor allele and minor allele frequency (MAF), nucleotide of SNP, *P* values of significant test and Bonferroni correction of *P* values.

Trait	SNP	Chr	Position (bp)	Minor allele and MAF	Nucleotide (major/minor allele)	*P*	Bonferroni correction
Betaine	rs109862186	5	118,820,845	B (0.18)	T/C	7.63E-07	0.03
L-Alanine	rs81117935	11	54,765,154	A (0.45)	T/C	9.10E-07	0.04
L-Lactic acid	rs42009425	22	41,109,447	A (0.19)	A/G	9.94E-07	0.04

**TABLE 4:** 200-kpb regions around the significant SNPs: chromosome (Chr), position of the region on chromosome (bp), gene in the regions and the location of the gene compared to SNP location.

Trait	Chr	Position (bp)	Gene name	Gene location compared to SNP location
Betaine	5	118,720,845–118,920,845	–	–
L-Alanine	11	54,665,154–54,865,154	*CTNNA2*	SNP is within gene
L-Lactic acid	22	41,009,447–41,209,447	–	–

In addition to the identified significant SNPs, the WssGBLUP method also identified more genomic regions associated with heritable metabolites based on additive genetic variance explained by SNP windows of 10 consecutive SNPs. The proportion of additive genetic variance explained by top 10 SNP windows and genes mapped in these windows are shown in Supplementary Table S1. The SNP window (107,403,824–107,704,991 bp) located on chromosome 5 was found to be associated with citric acid and explained the highest proportion of additive genetic variance (4.21%) while the SNP window (35,619,632–36,428,58 bp) with the lowest proportion of additive genetic variance (0.62%) was located on chromosome 26 and associated with L-lactic acid. A total of 368 unique genes were identified within the selected SNP windows (Supplementary Table S1). Further, five SNP windows showed pleiotropic effects on two or more metabolites and were mapped to 17 candidate genes (Table 5).

**TABLE 5:** Chromosome (Chr) and position of overlapped windows (bp) and genes in the overlap windows.

Traits	Chr	Position (bp)	Gene name
Acetone, L-lactic acid	1	28,675,718–29,049,389	*GBE1*
L-Alanine, choline	7	13,336,301–13,632,174	*IER2, STX10, TRMT1, LYL1, NACC1, NFIX, CACNA1A*
L-Alanine, betaine	19	24,357,241–24,917,540	*RAP1GAP2, SPATA22, OR1G1, ASPA, TRPV1, TRPV3*
L-Alanine, creatine	21	49,290,972–49,623,230	*GEMIN2, PNN*
Creatine, choline	28	15,916,594–16,124,333	*ANK3*

**FIGURE 5:**
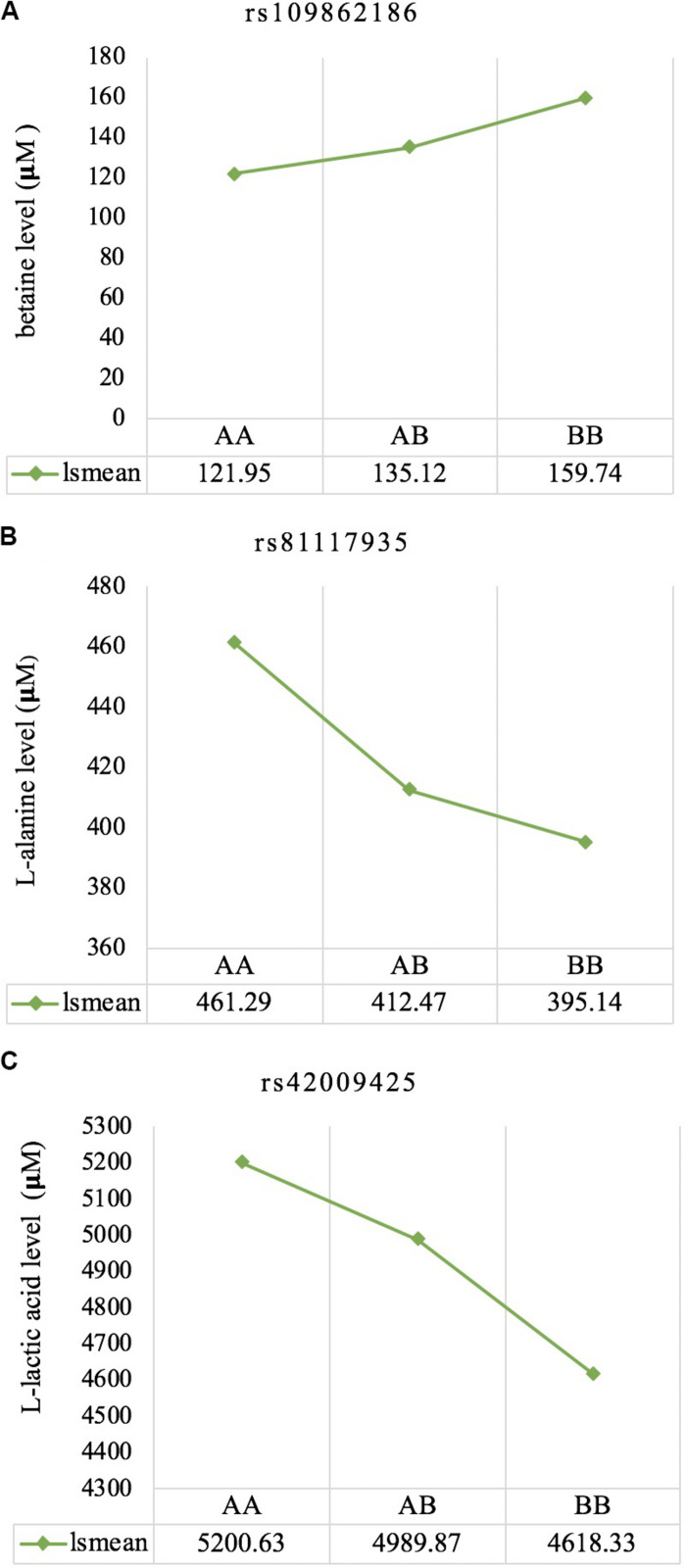
Least square means for the genotypic classes of significant SNPs associated with betaine **(A)**, L-alanine **(B)**, and L-lactic acid **(C)**, respectively. All three significant SNPs (rs109862186, rs81117935, and rs42009425) showed characteristics of additivity with the associated metabolite.

The least square means of the genotypic classes are given in Figure 5. All three significant SNPs (rs109862186, rs81117935, and rs42009425) showed characteristics of additivity with the associated metabolite as concentration either increased or decreased with the number of “B” alleles for the three genotypic classes.

### Functional Enrichment Analyses

**FIGURE 6:**
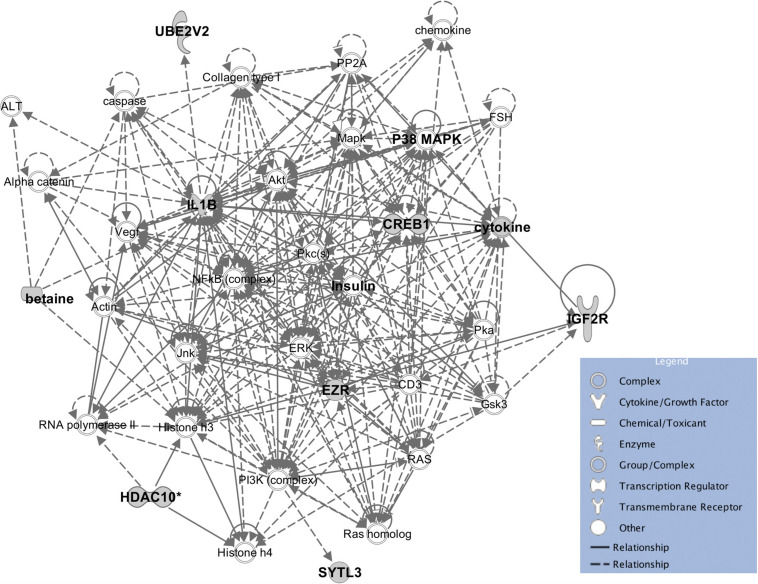
The enrichment network for betaine and associated genes, and the molecules in IPA database. The enriched pathway predicted by IPA showed a potential relationship between betaine, insulin, and phospholipids.

The eleven heritable metabolites and their candidate genes were significantly enriched (*P* < 0.05) for biological functions related to cellular, tissue, and organ development, cell-to-cell signaling and interaction, molecular transport, small molecule biochemistry, lipid metabolism, carbohydrate metabolism, and cellular growth and proliferation. All significant biological functions and their *P* values for each metabolite are provided in the Supplementary Table S2. Additionally, the IPA software produced 33 networks with the input metabolite and candidate gene lists (Supplementary Table S3) and one of the most informative networks (Figure 6) was related to lipid metabolism and cell-to-cell signaling and interaction with betaine and some of its candidate genes.

## Discussion

### Heritability Estimates

Metabolites have the potential to serve as biomarkers for production traits and diseases in livestock (Montgomery et al., 2009), and the concentration of biomarkers should not vary too much over the short term within an individual because such variation could undermine the predictive association in a single sample (Nicholson et al., 2011b). Most highly conserved metabolites are also highly heritable (Yousri et al., 2014) and less influenced by the environmental changes. In this study, we performed a baseline investigation into the heritability of plasma metabolites in crossbred beef cattle and identified potential associations between heritable metabolites and SNP markers. As certain metabolites are essential for growth and health, knowledge of the genetic parameters of these important metabolites could trigger directional selection toward regulating their concentration in metabolic processes. For instance, alanine is an essential amino acid for T cell activation (Ron-Harel et al., 2019) which affects immunity level. Here, a total of 11 metabolites out of 33 showed low to moderate heritability, suggesting their potential as biomarkers for genetic selection. Betaine and choline which showed moderate heritability in this study have been previously identified to be associated with residual feed intake in beef cattle (Karisa et al., 2014), thus, they could potentially be used as biomarkers for improving feed efficiency in beef cattle. The majority of the metabolites with negligible heritability may be largely influenced by environmental effects such as age, gender, nutrition, medication, and possibly underlying diseases (Beuchel et al., 2019). The non-heritable status of these metabolites may be used as a guide to animal management. For example, ruminants fed silage-based diets are likely to ingest ethanol because of ethanol production in fermented feeds (Nishino and Shinde, 2007) and the process of ethanol detoxification in liver could affect splanchnic nutrient metabolism (Obitsu et al., 2013). Ethanol showed a negligible heritability in this study, which suggests that the variation of ethanol concentration may be mainly affected by management factors such as feed.

In a related study that utilized milk metabolites from dairy cattle, Buitenhuis et al. (2013) found heritability estimates that were similar to estimates observed for five metabolites from the current study. Although, these studies are not completely comparable, this finding corroborates the possible existence of a genetic basis for plasma metabolites. In addition, the negligible heritability or large standard error observed for some of the metabolites may be due to the limited number of animals utilized. Thus, further study may be warranted as this is the first attempt to characterize the genetic basis of plasma metabolites in crossbred beef cattle.

### SNP Association, Candidate Genes and Genetic Effects

Genetic profiling of plasma metabolites has been previously studied in other species to assess their value as biomarkers for disease prediction (López-López et al., 2018). Recently, metabolomics GWAS was performed to identify genomic regions associated with variation in milk metabolites in dairy cattle (Buitenhuis et al., 2013). To the best of our knowledge, this study is the first attempt at profiling the genetic basis of plasma metabolites in crossbred beef cattle. The SNPs and candidate genes identified here revealed the potential association between metabolomics and genetics, which could help fill the knowledge gap that exist between genetic level and external phenotype. The possible signals detected in this study were associated with betaine, L-alanine and L-lactic acid, and the peaks for significant additive SNPs including rs109862186, rs81117935, and rs42009425 were on chromosome 5, 11, and 22. Two of the SNPs rs109862186 and rs42009425 showed no evidence of a candidate gene within 200-kbp distance, however, SNP rs42009425 associated with L-lactic acid was reported to be associated with clinical mastitis in French Holstein cattle (Marete et al., 2018). The SNP rs81117935 associated with L-alanine was found to be located within the candidate gene *CTNNA2* which is one of three human alpha-catenin genes. Alpha-catenin functions as a linking protein between cadherins and actin-containing filaments of the cytoskeleton (Cooper and Hausman, 2000), however, it is not known whether *CTNNA2* gene may regulates the concentration of L-alanine in bovine blood. The least square mean results (Figure 5) showed that the concentration of L-alanine was significantly (*P* < 0.05) greater in individuals that are homozygotes for the “A” allele of SNP rs81117935 while no significant differences existed for the other two genotypic classes. Our finding suggests that *CTNNA2* gene may play a role in the regulation of plasma L-alanine which requires further investigation.

Further, several candidate genes associated with heritable metabolites were mapped inside the selected SNP windows of 10 consecutive SNPs based on WssGBLUP analyses. Here, *choline kinase alpha* gene (*CHKA*) which is associated with choline was mapped inside the SNP window (46,143,465–46,796,930 bp) on chromosome 29. This gene encodes an enzyme called choline kinase alpha (Hosaka et al., 1992) which catalyzes the phosphorylation of choline to phosphocholine (Aoyama et al., 2004) as a first step in the biosynthesis pathway of phosphatidylcholine (Lacal, 2001). Phosphatidylcholine is one of the most abundant phospholipids in all mammalian cell membranes (van der Veen et al., 2017) and plays a critical role in membrane structure and also in cell signaling (Lacal, 2001). The importance of phospholipid metabolism in regulating lipid, lipoprotein and whole-body energy metabolism has been reviewed by van der Veen et al. (2017). Lipid metabolism has been previously identified as an important biological function in relation to beef cattle residual feed intake (Chen et al., 2011; Alexandre et al., 2015; Mukiibi et al., 2018). Therefore, the relationship between *CHKA* gene and choline metabolite used in this study have potential value for improving feed efficiency in beef cattle. Interestingly, several overlapped SNP windows were also identified, which indicates that either two metabolites were controlled by the same gene or by different genes within a SNP window (Table 5). The substantial polygenic and pleiotropic nature of the metabolite variation observed in the current study have been previously reported in human metabolomics studies (Hu et al., 2018; Gallois et al., 2019).

Several reasons may lead to the few significant SNPs identified. Firstly, variation in metabolite concentrations may be due to the polygenic nature of the genes underlying the variation. Polygenic inheritance for primary metabolites have been reported in plants (Rowe et al., 2008; Chan et al., 2010; Wen et al., 2014) and could potentially exist in beef cattle as evident in our study that utilized primary metabolites. Secondly, the crossbred nature of our studied population could lead to inconsistent linkage disequilibrium across multiple populations (De Roos et al., 2009). Thirdly, the ability to identify SNPs and quantitative trait loci with large effects on any of the metabolites depends partly on the amount of variation in metabolite concentration that can be attributed to genetic source. Here, low to moderate heritability were observed for some of the metabolites studied. Marker density is another factor that may lead to identification of fewer significant SNPs associated with variation in metabolites. In this study, 50K SNP panel was used for mGWAS, however, some causative SNPs may not be included in this panel and thus, would likely not be detected. Studies involving other beef cattle traits have shown that increasing marker density from 50K to 7.8 million SNPs can capture more additive genetic variance and can detect additional or novel significant SNPs (Wang et al., 2020; Zhang et al., 2020). Therefore, high-density SNP marker panel or whole-genome sequence data are suggested for future studies. Lastly, a stringent significance threshold based on Bonferroni correction for multiple testing was imposed to identify significant SNPs and exclude false positive results. However, compared with traditional GWAS, metabolites are highly correlated to other similar metabolites and often cannot be considered as independent. The traditional multiple testing methods may therefore eliminate some valuable SNPs. Some groups have computed the Bonferroni correction by counting all the metabolites (Gieger et al., 2008; Illig et al., 2010; Suhre et al., 2011), while a few other groups have adopted a less stringent strategy by taking into account the number of independent metabolites as determined by a principal component analysis to adjust for multiple test correction (Demirkan et al., 2012).

### Functional Enrichment Analyses

A one-to-one metabolite-to-gene correspondence is not known *a priori* (Nicholson et al., 2011a) but functional enrichment analyses could provide enriched functions and networks of metabolites and identified candidate genes to give a whole picture of gene-metabolite associations. Some biological functions that are significantly enriched may help us improve understanding of molecular factors for some important traits, such as feed efficiency. The eight most significantly enriched biological functions for beef cattle feed efficiency included lipid metabolism, amino acid metabolism, carbohydrate metabolism, energy production, molecular transport, small molecule biochemistry, cellular development, and cell death and survival (Cantalapiedra-Hijar et al., 2018). Our results supplement the part played by genetic and molecular factors for these functions, thus, available data with both information (i.e., metabolite data and feed efficiency related traits) could be used to elucidate this hypothesis. Detailed insight into the specific pathways that are affected by variation in metabolites is a useful first step to select the most likely hypotheses. A good example is betaine which is widely distributed within the animal body (Xia et al., 2018) and was reported to enhance the synthesis of methylated compounds such as phospholipids as well as directly influence lipid metabolism (Huang et al., 2008). In addition, a recent study showed that insulin was associated with phospholipid alterations, but the mechanism is still not clear (Chang et al., 2019). Interestingly, the enriched pathway constructed by IPA showed a relationship between betaine, insulin and phospholipids and provides new insight into the connection between them (Figure 6), however, this connection requires experimental validation.

## Conclusion

This study estimated heritability of 33 plasma metabolites for crossbred beef cattle and found low to moderate heritability for 11 metabolites, which provides evidence for the genetic basis underlying the variation of metabolite concentrations. Three significant SNP associations were detected for betaine (rs109862186), L-alanine (rs81117935), and L-lactic acid (rs42009425) which suggest that the genetic effects may be largely polygenic. The SNP rs81117935 was found to be within *CTNNA2* gene which is possibly associated with the regulation of L-alanine concentration in bovine blood. Other candidate genes were identified based on additive genetic variance explained by SNP windows of 10 consecutive SNPs. The observed heritability estimates and candidate genes and networks identified in this study will serve as baseline information for further research into the utilization of plasma metabolites for genetic improvement of crossbred beef cattle.

## Data Availability Statement

The SNP data for this study have been submitted to the UAL Dataverse at https://doi.org/10.7939/DVN/8X1YZC.

## Ethics Statement

All the animals were managed and cared for according to the guidelines of the Canadian Council on Animal Care, CCAC (Olfert et al., 1993).

## Author Contributions

JL carried out data analyses and initiated, drafted, and revised the manuscript with help from GP, EA, and TV. EA helped to manage quality control of data and construct statistic models. TV helped with single-step genomic BLUP analyses. MA-I helped with candidate gene mapping and functional enrichment analyses. BK designed the primary study, helped with data collection and provided information on animals, blood samples, and NMR spectra in the materials section. ZW contributed to the statistics and genetics background of the study. GP was the principal investigator of the project, participated in project management, and experimental design. All authors read and approved the final manuscript.

## Conflict of Interest

The authors declare that the research was conducted in the absence of any commercial or financial relationships that could be construed as a potential conflict of interest.

## Footnotes

1 http://www.beefbooster.com

2 www.Ingenuity.com
